# The Role of a New Stabilizer in Enhancing the Mechanical Performance of Construction Residue Soils

**DOI:** 10.3390/ma17174293

**Published:** 2024-08-30

**Authors:** Xin Chen, Jing Yu, Feng Yu, Jingjing Pan, Shuaikang Li

**Affiliations:** 1 Institute of Foundation and Structure Technologies, Zhejiang Sci-Tech University, Xiasha Higher Education Park, Hangzhou 310018, China; 202110301002@mails.zstu.edu.cn (X.C.); yujing981140@126.com (J.Y.); 2 School of Civil Engineering and Architecture, Zhejiang Sci-Tech University, Xiasha Higher Education Park, Hangzhou 310018, China; jingjingpan@zstu.edu.cn; 3 Zhejiang Engineering Construction Management Co., Ltd., West Lake District, Hangzhou 310016, China; zjjhlsk317@163.com

**Keywords:** construction residual soil, industrial waste-based soil stabilizer, mechanical properties, water stability, dry–wet cycle

## Abstract

Urban construction generates significant amounts of construction residue soil. This paper introduces a novel soil stabilizer based on industrial waste to improve its utilization. This stabilizer is primarily composed of blast furnace slag (BFS), steel slag (SS), phosphogypsum (PG), and other additives, which enhance soil strength through physical and chemical processes. This study investigated the mechanical properties of construction residue soil cured with this stabilizer, focusing on the effects of organic matter content (*O*_o_), stabilizer dosage (*O*_c_), and curing age (*T*) on unconfined compressive strength (UCS). Additionally, water stability and wet–dry cycle tests of the stabilized soil were conducted to assess long-term performance. According to the findings, the UCS increased with the higher stabilizer dosage and longer curing periods but reduced with the higher organic matter content. A stabilizer content of 15–20% is recommended for optimal stabilization efficacy and cost-efficiency in engineering applications. The samples lost their strength when immersed in water. However, adding more stabilizers to the soil can effectively enhance its water stability. Under wet–dry cycle conditions, the UCS initially increased and then decreased, remaining lower than that of samples cured under standard conditions. The findings can provide valuable data for the practical application in construction residual soil stabilization.

## 1. Introduction

With the rapid urbanization and modernization of China, the strategic development and efficient utilization of underground spaces have become increasingly important. However, the construction of underground tunnels and excavation projects generates substantial amounts of construction residue soil, posing a critical challenge to the low-carbon development of underground engineering projects [1]. Traditional methods for disposing of construction residue soil, including piling, landfilling, and backfilling, have presented numerous disadvantages [2]. Piling and landfilling consume significant land resources and pose risks of secondary environmental pollution [3]. Additionally, backfilling is limited by specific project requirements and the characteristics of the waste material [4]. Chemical stabilization treatment can effectively address the challenges of construction residue soil disposal and significantly enhance its comprehensive utilization rate [5]. Stabilized soil can be utilized as engineering fill and applied in subgrade and foundation engineering projects. This application not only reduces the demand for natural materials but also promotes resource recycling.

However, factors such as engineering type, geological variations, and construction methods lead to differences in the water content and organic matter of construction residue soil. The properties of construction residue soil directly impact the stability and safety of engineering projects. Therefore, the mechanical characteristics of stabilized soil must be carefully considered in engineering applications. Currently, numerous researchers have explored the mechanical properties of stabilized soil and yielded significant results. Pan et al. [6] developed a stabilizer through a mix design for reinforcing silt and found that the strength of the stabilized soil increased with an increase in stabilizer dosage (*O*_c_) and curing age (*T*). Cao et al. [7] explored the impact of several variables, such as initial water content, cement dosage, organic matter content (*O*_o_), curing temperature, and curing period, on the unconfined compressive strength (UCS) of cemented soil. The findings revealed that UCS decreases with increasing *O*_o_ and initial water content, whereas it increases with higher temperatures and cement dosage. Oliveira and Reis [8] investigated the influence of *O*_o_ on the compressibility, stiffness, and strength of xanthan gum-solidified soil. They found that when the *O*_o_ of the stabilized soil ranges from 1.5% to 5.5%, its strength initially rises and then falls. Mohammed et al. [9] investigated the effects of soil strength on *O*_o_, *O*_c_, and *T* using sewage sludge as a means of controlling the soil’s organic matter content. The results indicated that strength loss occurred in an increase in *O*_o_.

Traditionally, cement has been widely used in soil stabilization. However, its production process results in significant carbon dioxide emissions, which has drawn widespread societal concern [10,11,12,13]. Moreover, industrial waste materials have been explored for their environmental friendliness, low cost, and cementitious potential [14]. Numerous scholars have studied their mechanical properties in soil stabilization [15,16,17,18]. Wattez et al. [19] investigated the performance of blast furnace slag (BFS) and cement mixed for stabilizing tunnel excavation slurry and verified its feasibility. Bian et al. [20] investigated the UCS of dredged soil stabilized with cement and phosphogypsum (PG) through UCS tests and found that PG significantly enhances the UCS of soils with different plasticity indexes, especially in low-plasticity soils. Luo et al. [21] demonstrated the feasibility of riverside soft soil treatment with a combination of fly ash, slag, and alkaline activators. According to the findings, the UCS cured for 28 days is 1.39 times greater than the cement-stabilized soil. Zheng et al. [22] verified the feasibility of a mixture of lime, granulated blast furnace slag (GGBS), PG, and soil as a feasible embankment fill material. Kumar and Munisingh [23] found that the optimal compressive strength for road construction fill material is achieved with a mixture ratio of fly ash, BFS, and cement at 20:3:2.

The aforementioned studies show that the incorporation of industrial waste materials not only improves the performance of stabilized soils but also promotes resource reuse, reduces environmental pressures, and provides crucial technical support for the resource utilization of industrial waste [24,25,26].

Therefore, the authors have independently developed a novel industrial waste-based soil stabilizer by mixing PG with BFS, steel slag (SS), and a small amount of cement. BFS and SS are by-products generated from the ironmaking and steelmaking processes, respectively. BFS primarily consists of dicalcium silicate (C_2_S), and the main components of SS include both C_2_S and tricalcium silicate (C_3_S). PG is an industrial solid waste produced during wet process phosphoric acid production, primarily composed of CaSO_4_·0.5H_2_O and CaSO_4_·2H_2_O. The new stabilizer undergoes a series of hydration and ionic reactions to form calcium silicate hydrate (C-S-H) gel and ettringite (AFt). This process improves the bonding strength between soil particles and the structural density [27].

However, the abundant groundwater and humid climate in the Hangzhou area presently serve as challenges to the long-term performance of stabilized soil. Prolonged exposure to a moist environment may reduce the durability of the stabilized soil and shorten its service life. In areas with high groundwater levels, the prolonged submersion of stabilized soil in water may lead to softening and cracking. Therefore, the dry–wet cycle tests and water stability tests are essential to conduct the verification of the durability and stability of stabilized soil under long-term service conditions [28,29,30]. MolaAbasi et al. [31] verified the feasibility of using zeolite for cement-stabilized sandy soil through the dry–wet cycle and UCS tests, revealing that zeolite reduces cumulative mass loss and enhances the strength and durability of stabilized soil. Wang et al. [32] investigated the performance of SS-stabilized soil for highway subgrades using UCS, compaction, CBR, and water stability tests, and confirmed its feasibility through field tests. Zhang et al. [33] proposed using lignin to stabilize silt and assessed its durability under various harsh conditions using UCS, water stability coefficient, and mass loss evaluations under soaking and dry–wet cycles. Niu et al. [34] obtained the optimal scheme for soil stabilization through direct shear tests and investigated its durability and stability via dry–wet cycle tests.

Although the new stabilizer showed excellent early strength, which has reached up to 5.58 MPa, which is higher than the early strength of cement soil at 2.79 MPa, studies on its mechanical properties and stability under long-term service conditions remain insufficient. Therefore, the purpose of this work is to investigate the effects of organic matter content (O_o_), stabilizer dosage (O_c_), and curing period (*T*) on soil strength after stabilization. Furthermore, the water stability test and dry–wet cycle test will be conducted to assess the durability of the newly stabilized soil. This research aims to establish a theoretical foundation for the potential application of stabilized construction residue soil in construction projects.

## 2. Materials and Methods

### 2.1. Materials

**Figure 1:**
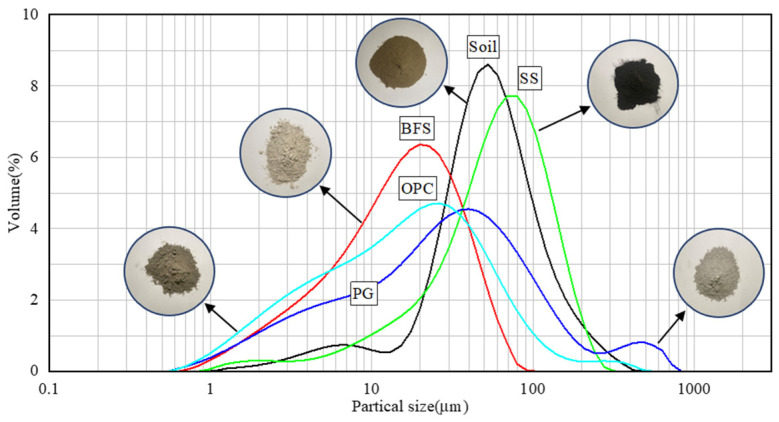
The particle size distribution curve of the soil sample and the raw materials of the stabilizer.

**Figure 2:**
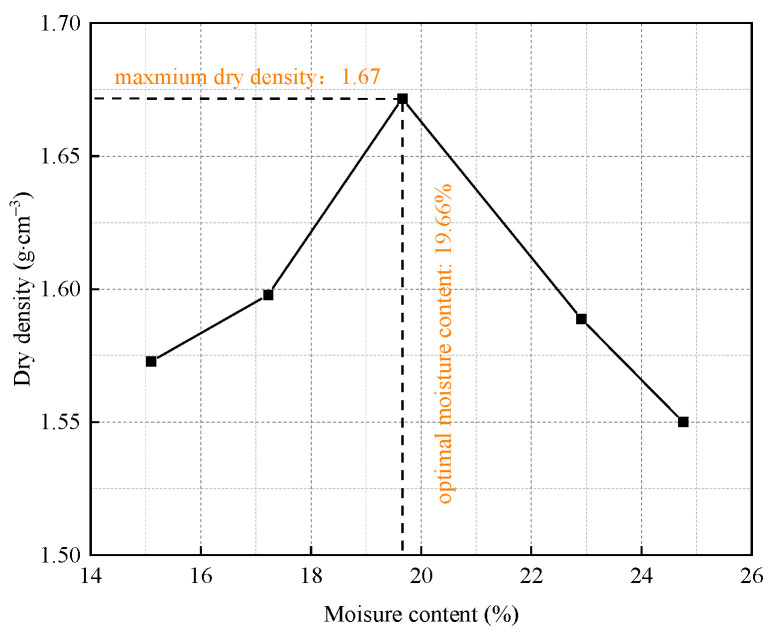
The compaction curve of the soil sample.

**Figure 3:**
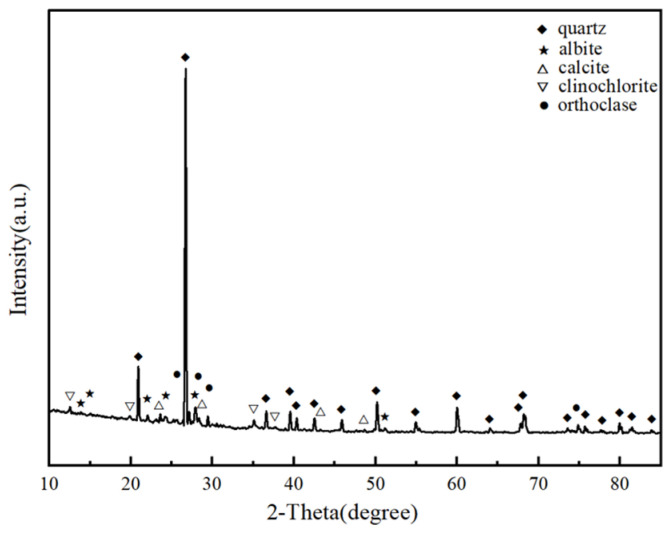
The mineral phase of tested soil.

The construction residue soil for testing was derived from a project in Hangzhou. Following this, the physical characteristics of the soil sample were ascertained by “Standard for geotechnical testing method (GB/T50123-2019)” [35] and presented in Table 1. The construction residue soil was classified as silt. Before the test, the raw residue soil utilized in this study was air-dried, and purified using a 2 mm screen. The particle size distribution curve and the compaction curve of the soil sample are shown in Figure 1 and Figure 2, respectively. The results indicated that the maximum dry density of the soil was 1.67 g/cm^3^, with an optimum moisture content of 19.7%. As can be seen in Figure 3, mineral-phase identification revealed the presence of quartz and albite within the soil sample. The chemical components of the soil sample and the main raw materials of the stabilizer are shown in Table 2.

The curing agent employed in this study is a novel soil stabilizer, whose physical properties have been detailed in Table 3. This innovative stabilizer is an eco-friendly cementitious material, formulated from industrial waste and polymer additives. When the stabilizer is mixed with soil, the strength and impermeability of the soil are significantly enhanced by a series of physical and chemical reactions.

### 2.2. Methods

**Figure 4:**
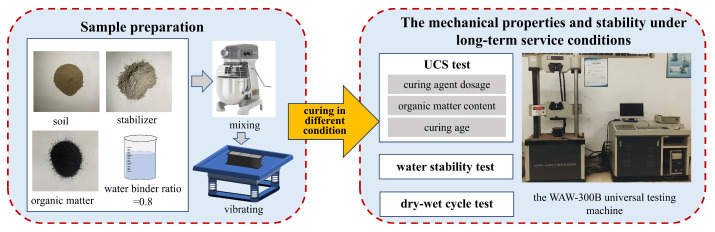
The methods of this study.

The method of this study is shown in Figure 4.

#### 2.2.1. Sample Preparation

To obtain wet soil, the screened silt is mixed with water at the optimum moisture content. The mass of the organic matter, wet soil, curing agent, and water were measured and thoroughly mixed by the experimental design. The mixture was filled into cubic molds of 70.7 × 70.7 × 70.7 mm. The samples were vibrated on a shaking table, covered with plastic film, and allowed to cure for 24 h before being demolded. Following demolding, the samples were tagged and allowed to cure in different conditions for the specified curing duration. For each group, three specimens were prepared to guarantee the accuracy of the experimental results.

#### 2.2.2. UCS Test

To investigate the influence of the new soil curing agent dosage (*O*_c_), organic matter content (*O*_o_), and curing time (*T*) on the mechanical properties of stabilized construction residue soil, different dosages of the curing agent (*O*_c_ = 15%, 20%, 25%, 30%) and organic matter (*O*_o_ = 0%, 2%, 4%, 6%) were employed for soil solidification. The variables and experimental plan are listed in Table 4. In this study, the organic matter content was adjusted using humic acid, with a purity of no less than 90%.

Following the “Specification for mix proportion design of cement soil (JGJ/T 233-2011)” [36], the strength of the stabilized soil samples was tested for UCS using a WAW-300B universal testing machine (Zhejiang Tugong Instrument Co., Ltd. Shao xing, China) after different curing durations (T = 7, 14, 21, 28 days).

The upper plate of the machine was manually adjusted to ensure perfect contact with the sample’s upper surface before the UCS test. To guarantee continuous and uniform loading until the sample fails, the test loading rate was set at 0.8 kN·m/s. The test result was determined by taking the arithmetic mean of three parallel specimens. If one outlier is identified, the arithmetic mean of the remaining two specimens is calculated. In the case of two outliers, the test must be repeated. The UCS ($f_{cu}$) is calculated using the following formula:

(1) $$f_{cu}=\frac{P}{A},$$

where $f_{cu}$ is the UCS of the stabilized soil specimen, *P* is the maximum failure load of the specimen, and *A* is the cross-sectional area of the specimen.

#### 2.2.3. Water Stability Test

A water stability test was conducted to further assess the stabilized soil’s performance. Specifically, groups with varying curing agent contents of 20% and 30%, along with organic matter contents of 0% and 6% as shown in Table 3, were selected for evaluation. Six samples were prepared for each group. Three of these samples were subjected to standard curing for 28 days, after which they were immersed in water for durations of 1 day, 25 days, and 40 days, respectively. The remaining three samples were also subjected to standard curing for the same period and tested for the UCS alongside the immersed samples. Before testing, the height of each sample was measured using a vernier caliper. Subsequently, the strength residual coefficient (*K*_a_) was determined for the samples at different immersion times using the following formula:

(2) $$K_{a}=\frac{f_{a}}{F_{28d}},$$

where $K_{a}$ is the strength residual coefficient, $f_{a}$ is the UCS after different immersion times or the UCS after each cycle, and $F_{28d}$ is the UCS after standard curing for 28 days.

#### 2.2.4. Dry–Wet Cycle Test

Meanwhile, the groups chosen for the dry–wet cycle test were the same as the water stability test. After being cured for 28 days, the test group was subjected to dry–wet cycles, while the control group remained in standard curing conditions. Five cycles were performed to evaluate the durability of the geotechnical material, with each cycle consisting of drying and wetting stages.

The test specimens were dried for 12 h in an oven with a temperature of 70 ± 2 °C. After that, the specimens were taken out of the oven and given an hour to return to room temperature before their mass was measured. Subsequently, the samples then underwent an 11 h immersion in water at 20 ± 2 °C to complete a single 24 h dry–wet cycle. After completing five cycles, UCS tests were performed on the test and control groups to determine the durability. Observations and measurements of the mass and size changes in the test samples were recorded after each cycle. Finally, the cumulative mass loss rate *C* was calculated with the formulas below.

(3) $$C=\sum_{i=1-j}\frac{W_{i}}{M_{0}}\left(j=1,2,3,4,5\right),$$

where *C* is the cumulative mass loss rate, $W_{i}$ is the mass loss after the *i*-th cycle, and $M_{0}$ is the initial mass of the sample before the cycle.

## 3. Experimental Results

### 3.1. UCS

#### 3.1.1. The Influence of Curing Agent Dosage on the UCS of Stabilized Soil

**Figure 5:**
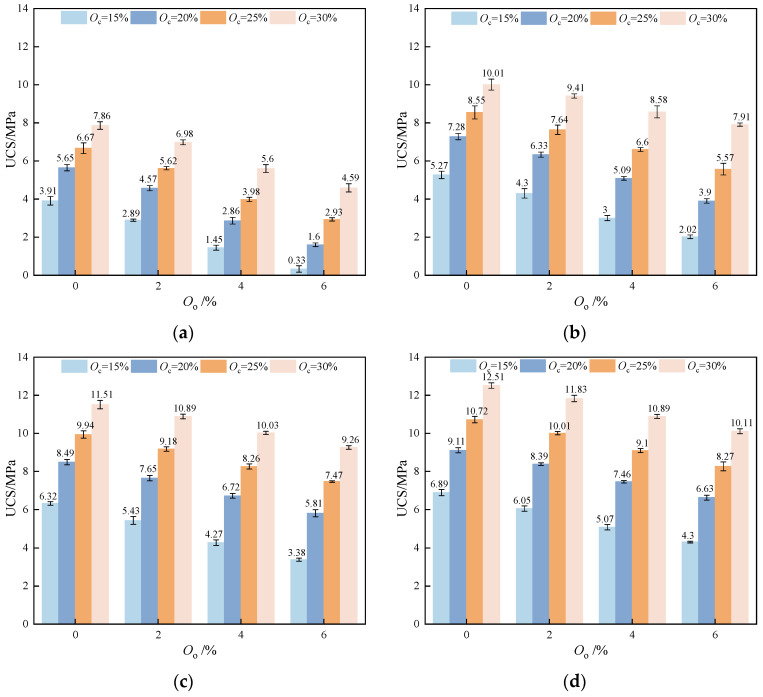
The relationship between the UCS of stabilized soil and the dosage of the stabilizer: (**a**) *T* = 7 d; (**b**) *T* = 14 d; (**c**) *T* = 21 d; (**d**) *T* = 28 d.

Figure 5 shows the relationship between the UCS of stabilized soil and stabilizer dosage. The results indicated a positive correlation between UCS and stabilizer content under the same curing age and organic matter content. According to the previous findings, an increase in stabilizer dosage led to a corresponding increase in UCS. This phenomenon can be explained by the increased formation of hydrates such as C-S-H gel and AFt as the stabilizer dosage increases. These hydration products play a critical role in binding soil particles and filling interstitial voids, resulting in increased soil density and strength. Moreover, Figure 5 reveals that the relationship between stabilizer dosage and UCS was not strictly linear. The strength growth curve of stabilized soil showed distinct phases, which can be categorized into active and inert intervals.

Table 5 shows the UCS growth rate for various stabilizer dosages. For example, with an organic matter content of 0%, the UCS growth rate reached 44.50% when the stabilizer content increased from 15% to 20% after 7 days of curing. In contrast, with stabilizer contents ranging from 20% to 25% and 25% to 30%, the growth rates were significantly lower, only 18.05% and 17.84%, respectively. This growth pattern was consistent with a 28-day curing age. Additionally, when the organic matter content rises, the growth rate of UCS with a stabilizer content ranging from 15% to 20% was superior to other dosage ranges. This suggested that when the stabilizer content exceeded 20%, the strength growth entered an inert phase. To optimize both the stabilizing effectiveness and cost-efficiency in engineering applications, a stabilizer content of 15–20% is recommended.

#### 3.1.2. The Influence of Organic Matter Content on the UCS of Stabilized Soil

**Figure 6:**
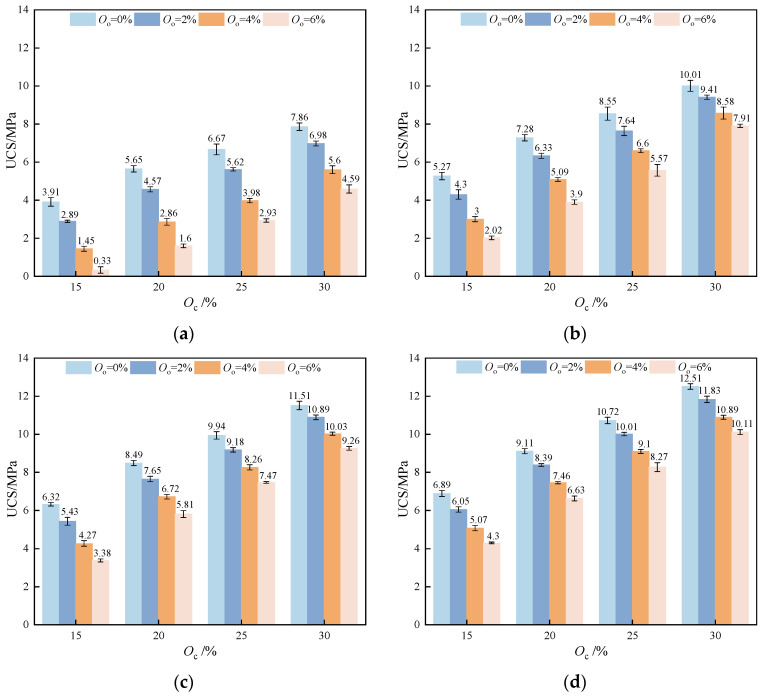
The variation in strength performance of stabilized soil with different organic matter contents: (**a**) *T* = 7 d; (**b**) *T* = 14 d; (**c**) *T* = 21 d; (**d**) *T* = 28 d.

Figure 6 shows the variation in the strength performance of stabilized soil with different organic matter contents. As shown in Figure 6, under the same curing age and the same amount of curing agent, the UCS of the stabilized soil decreased with the increased organic matter content. To quantify this variation, the strength loss rate was defined. The strength loss rate is the ratio of the difference in UCS between stabilized soil with 0% organic matter and that with various organic matter contents to the strength of the specimen when the content of organic matter is 0%. The formula is expressed as follows:

(4) $$S=\frac{\left|F_{0\%}-\left.F_{x}\right|\right.}{F_{0\%}}\times 100\%,$$

where S is the strength loss rate of stabilized soil, $F_{0\%}$ is the UCS of stabilized soil with 0% organic matter content, and $F_{x}$ is the UCS of stabilized soil with other organic matter contents.

Table 6 shows the changes in the strength loss rate as organic matter content increases. When the dosage of the curing agent was 15%, the strength of the specimen with 2% organic matter content was 26.09% lower than that of the specimen with 0% organic matter at *T* = 7 d. After being cured for 14, 21, and 28 days (*T* = 14, 21, 28 d), the former was reduced by 18.41%, 14.08%, and 12.19%, respectively, compared to the latter. Comparing the strength loss rates of other contents, the influence of organic matter content was particularly significant at *T* = 7 d and gradually weakened with longer curing periods. Moreover, the impact of organic matter on the strength was alleviated with an increase in the stabilizer dosage. The hydration mechanism primarily involves hydration and pozzolanic reactions, which produce cementitious materials such as C-S-H and AFt crystals that fill the pores. In this study, the amount of acidic humic acid rose along with the amount of organic matter. The abundance of H^+^ in the system leads to the consumption of OH^−^, thereby inhibiting the hydration and pozzolanic reactions of the curing agent. The generation of essential hydration products is reduced due to this inhibition. Consequently, the stabilized soil’s strength is significantly reduced as fewer hydration products are formed, which are vital for the integrity of the stabilized soil.

#### 3.1.3. The Influence of Curing Age on the UCS of Stabilized Soil

**Figure 7:**
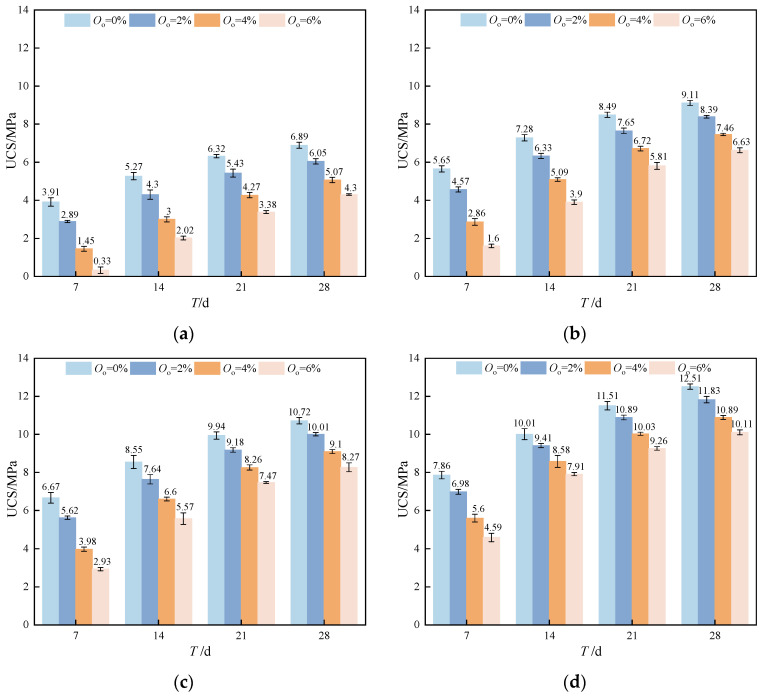
The relationship between the UCS of stabilized soil and the dosage of the stabilizer: (**a**) *O*_c_ = 15%; (**b**) *O*_c_ = 20%; (**c**) *O*_c_ = 25%; (**d**) *O*_c_ = 30%.

The differences in stabilized soil’s UCS at various curing ages are shown in Figure 7. The strengths of the stabilized soil cured at 7 days, 14 days, 21 days, and 28 days were 5.65 MPa, 7.28 MPa, 8.49 MPa, and 9.11 MPa, respectively, when the stabilizer dosage was 20% and the organic matter content was 0%. This pattern suggested that as the curing period increased, the UCS of stabilized soil grew. Due to the restricted hydration product development in the early curing phases, the connections of soil particles are relatively weak and the strength is reduced. However, as the curing period extends, the unreacted active substances in the stabilizer become further activated, leading to the production of more hydrates. These products gradually develop and fill the soil pores, forming a dense structure that improves the soil’s strength.

The strength growth rate of stabilized soil with curing age is displayed in Table 7. According to the results, the UCS of the stabilized soil grew by 28.85% from 7 to 14 days, by 16.62% from 14 to 21 days, and by just 7.30% from 21 to 28 days with a stabilizer content of 20% and an organic matter content of 0%. This demonstrated that the new stabilizer soil exhibited high early strength, with the growth rate diminishing over time. The stabilized soil showed poor strength initially when the organic matter content was high (*O*_o_ = 6%), but it increased rapidly from 7 to 14 days, reaching 512.12% (*O*_c_ = 15%). Additionally, the growth rate remained significant at 27.22% between 21 and 28 days. The phenomenon was speculated to be due to the presence of H^+^ in the organic matter, which initially inhibits the formation of an alkaline environment by OH^−^, leading to lower early strength. However, as the curing period grows, the H^+^ in the organic matter is gradually consumed, allowing the stabilizer to become activated in the alkaline environment. This activation leads to hydration reactions that generate cementing substances, which fill the soil pores and improve the strength at later stages.

Moreover, compared to the previous research, this study demonstrated the significant advantages in both early and later strengths using a novel stabilizer, as shown in Table 8. When the stabilizer content was 20%, the UCS of stabilized soil after 28 curing days in this study was 9.11 MPa, surpassing the 28-day curing performance of the GS stabilizer reported in reference [37]. Furthermore, the novel stabilized soil presented in this study exhibited a good early curing effect, achieving a 7-day curing period UCS of 5.65 MPa. The value is significantly higher than the UCS cured for 7 days reported in the literature [38], which ranges from 1.2 to 1.6 MPa. Dong et al. [39] employed a 25% stabilizer content for stabilizing soft soil, resulting in a 28-day curing period UCS of 8.85 MPa, which is lower than the strength of 9.11 MPa observed in this study. The novel stabilizer also exhibited superior performance when the soil contained organic matter. With the same stabilizer content, the stabilized soil with 4% organic matter content in this study achieved a 7-day curing period strength of 2.86 MPa and a 28-day curing period UCS of 7.46 MPa, both greater than the corresponding strength reported in the literature [7]. When the organic matter content was 6%, the 28-day curing period UCS of the novel stabilized soil reached 4.30 MPa, which significantly exceeded the strength reported in reference [40].

### 3.2. Water Stability Test

**Figure 8:**
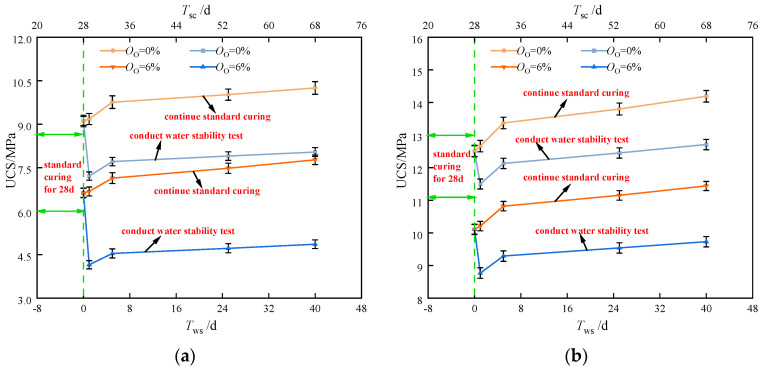
The variation of UCS of stabilized soil with immersion time: (**a**) *O*_c_ = 20%; (**b**) *O*_c_ = 30%.

Figure 8 displays the variation of UCS of stabilized soil with immersion time. In Figure 8, the immersion time of the samples after 28 days of standard curing ($T_{\mathrm{ws}}$) is shown by the lower horizontal axis, while the curing period of the control group under standard conditions ($T_{\mathrm{sc}}$) is represented by the upper horizontal axis. The strength of the specimen deteriorated during the immersion procedure when the contents of the organic matter and stabilizer were constant. This was evidenced by the UCS of the immersed specimens being significantly lower than those cured under standard conditions. This reduction in strength highlighted the detrimental impact of prolonged exposure to water on the structure of the stabilized soil. With the increasing curing age, more hydration products fill the pores between soil structures because of the thorough hydration reaction. This process enhances the water stability of the soil. Consequently, there is an increasing trend in the UCS of soil samples cured under standard conditions as well as those subjected to immersion.

**Figure 9:**
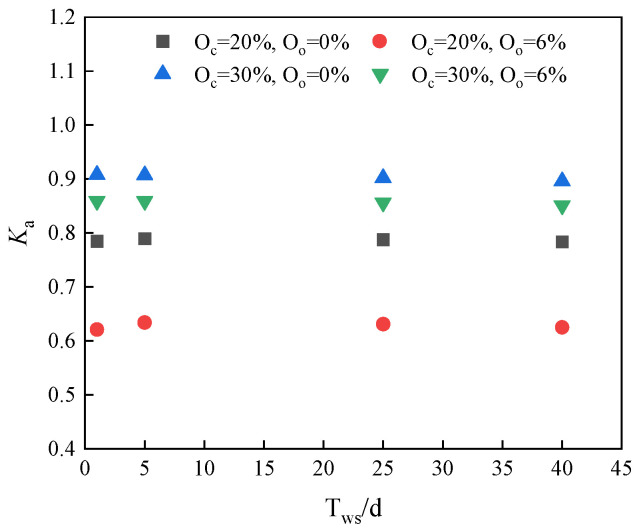
The strength residual coefficients of the new stabilized soil at different immersion periods.

Figure 9 shows the strength residual coefficients of the new stabilized soil at different immersion periods, as calculated by Equation (2). The results indicated that the strength residual coefficients of the stabilized soil samples varied from 0.62 to 0.91. The coefficient is closer to 1, indicating that less damage of the specimen was sustained from immersion. After the immersion test, the strength residual coefficient of stabilized soil samples with 20% curing agent and 0% organic matter content was around 0.8. In contrast, the samples with the same dosage of curing agent but with 6% organic matter exhibited a lower strength residual coefficient of approximately 0.6, indicating greater damage due to immersion. When the stabilizer content was increased to 30%, the strength residual coefficient with 6% organic matter ranged from 0.8 to 0.9. Notably, samples with the same dosage of curing agent and 0% organic matter demonstrated the highest strength residual coefficients, ranging from 0.9 to 0.95. This near-maximum retention of strength highlighted the effectiveness of a higher curing agent content in mitigating strength loss due to immersion. In summary, increasing the dosage of the stabilizer could enhance the strength residual coefficient of stabilized soil samples post-immersion, particularly in the absence of organic matter.

### 3.3. Dry–Wet Cycle Test

**Figure 10:**
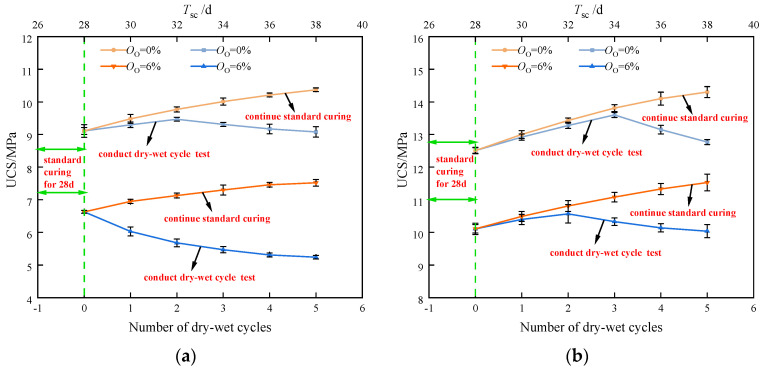
The UCS of the new stabilized soil varied with the wet–dry cycle numbers: (**a**) *O*_c_ = 20%; (**b**) *O*_c_ = 30%.

The UCS of newly stabilized soil samples was tested and compared with the control group following different numbers of the dry–wet cycles. Figure 10 displays the dry–wet cycle test results. In Figure 10, the number of dry–wet cycles for the test group is shown by the lower horizontal axis, while the curing duration for the control group under normal circumstances is represented by the upper horizontal axis. The number of dry–wet cycles and the UCS did not have a perfectly linear connection. When the cycles were fewer than three, the UCS of the stabilized soil samples grew as the cycle numbers increased. However, a reduction in UCS was observed beyond three cycles. Compared to the group cured under standard conditions, the UCS of the dry–wet cycle specimens was generally lower. This was due to the dry–wet cycle test comprising both drying and wetting phases. During the drying phase, the increased temperature and extended curing time led to the rapid formation of hydration products, which caused the development of microcracks in the stabilized soil. These abundant hydration products fill the soil pores and microcracks, resulting in an increment in the strength. Therefore, the influence of these microcracks on the effective stress was minimal after one to two cycles. However, after three cycles, the development of microcracks within the stabilized soil significantly affected the integrity and strength of the samples, causing the UCS to reduce with the increasing cycle number.

**Figure 11:**
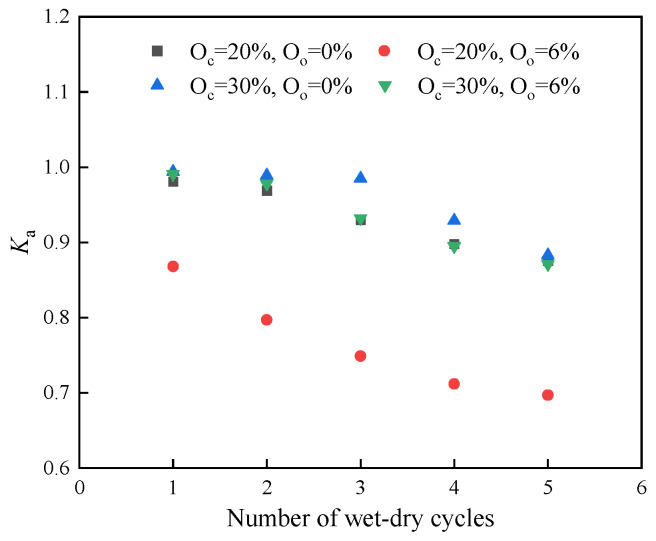
The residual coefficients of stabilized soil under wet–dry cycling conditions.

The strength residual coefficient is introduced to compare the UCS of specimens undergoing dry–wet cycles with the UCS of specimens cured under standard conditions. Figure 11 shows the residual coefficients of stabilized soil after dry–wet cycling. The coefficients of newly stabilized soil under different numbers of dry–wet cycles, as seen in Figure 11, were all smaller than 1, showing a significant deterioration in the strength attributed to dry–wet cycling. The strength residual coefficient of samples with 20% curing agent content and 6% organic content was 0.71 after five cycles. When the stabilizer content was increased to 30%, the strength residual coefficient after five cycles improved to 0.87. This improvement demonstrated that increasing the stabilizer content can effectively enhance the strength residual coefficient and resistance to dry–wet cycling of the stabilized soil.

**Figure 12:**
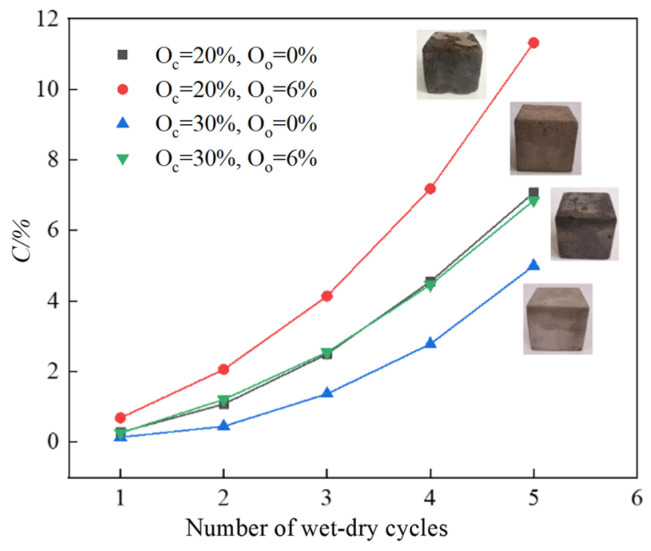
The cumulative mass loss rate varies with the cycle numbers.

Meanwhile, the mass of the specimens was recorded during the experiment. The cumulative mass loss rate varies with the cycle numbers as shown in Figure 12. Under dry–wet cycling conditions, the cumulative mass loss rate of stabilized soil rose with the increasing cycle numbers. After the third cycle, the impact of dry–wet cycling on the specimen’s mass became primarily significant. Before the third cycle, the cumulative mass loss rate of stabilized soil under dry–wet conditions showed a relatively gradual increase. Following the third cycle, microcracks develop in the soil due to shrinkage and swelling, which greatly accelerates the mass loss rate. When the organic content was 6%, the mass loss rate of samples with 30% curing agent content exhibited a lower mass loss rate compared to those with 20% curing agent content. This indicated that the mass loss of stabilized soil samples was positively impacted by the stabilizer dosage, consistent with the pattern observed in Figure 11.

## 4. Discussion

The experimental results confirmed the mechanical properties and stability of the novel stabilized soil under long-term use conditions. The industrial waste-based novel stabilizer used in this paper is mainly composed of BFS, SS, PG, and a small amount of cement [27]. The UCS of the stabilized soil increased with the increasing stabilizer content and curing age, following a pattern consistent with that of cement [30]. In the BFS–cement system, cement hydration primarily generates C-S-H gel and Ca(OH)_2_. BFS is primarily activated by Ca(OH)_2_ from cement hydration [41], producing mainly C-S-H gel, calcium aluminate hydrate (C-A-H) gel, and minor amounts of Ca(OH)_2_ [42]. Wu et al. (2020) [43] noted that SS created an alkaline environment conducive to BFS hydration and improved the workability and erosion resistance of the stabilized soil. The interaction between SS and water produces C-S-H, C-A-H, and Ca(OH)_2_ [44]. Moreover, the PG in the novel stabilizer reacts with Ca(OH)_2_ to form the AFt crystals, imparting strength to the stabilized soil [39]. The AFt crystals fill the pores within the soil, accelerating early strength development [45]. During this process, as the content increases, more C_2_S, C_3_S, and sulfate ions react in the stabilized soil system, leading to more hydration products and higher strength. Over time, the hydration reaction becomes increasingly complete.

However, the strength of the stabilized soil decreased with an increase in organic matter content. This finding is consistent with the trends reported by Ma et al. (2016) [40], Du et al. (2020) [46], and Cao et al. (2022) [7]. The organic matter used in this study is humic acid. The reduction in stabilized soil strength attributed to humic acid may be due to its particles being more compressible than other inorganic minerals present in the soil [47]. The humic acid particles are negatively charged, leading to their adsorption onto soil particle surfaces, which in turn affects the hydration reaction [47].

Moreover, environmental humidity and the service conditions of the stabilized soil are critical to its long-term performance. The water stability and wet–dry cycle tests demonstrated good durability, further validating that stabilized engineering waste soil can be effectively used as recycled fill material in subgrade construction. Nevertheless, this study has certain limitations. This study did not include an environmental impact assessment of the industrial waste-based stabilizer. In future research, we will integrate a microstructural analysis, leaching tests, and life cycle assessment to further evaluate the environmental performance of this stabilizer.

## 5. Conclusions

In this paper, the UCS, water stability, and dry–wet cycle resistance of stabilized soil with different stabilizer contents, organic matter contents, and curing periods have been studied. The conclusions are as follows:

(1) The UCS of stabilized soil grows with the stabilizer dosage and the curing period but decreases as the organic matter content increases. Moreover, the strength influenced by the organic matter content diminished with longer curing time. The UCS showed active and inert phases as the amount of stabilizer increases. To optimize both stabilization efficacy and cost-efficiency in engineering applications, a stabilizer content of 15–20% is recommended.
(2) Under the same stabilizer and organic matter content conditions, the UCS of the immersed specimens was significantly lower than those cured under standard conditions. Water immersion led to strength degradation in stabilized soil samples, with the residual strength coefficient ranging from 0.62 to 0.91. However, increasing the stabilizer content can significantly enhance its water stability.
(3) The UCS of the newly stabilized soil during dry–wet cycling exhibited an initial increase followed by a decrease with the increasing cycle numbers. However, its UCS remained lower than those cured under standard conditions. The residual strength coefficient of the stabilized soil was less than 1, indicating a marked deterioration in strength characteristics. Additionally, the cumulative mass loss rate increased with dry–wet cycle numbers.

In conclusion, the new stabilizer-treated construction residue soil exhibits excellent compressive strength and durability and can be applied as recycled filler in subgrade engineering and excavation engineering, thereby satisfying the requirements of engineering construction.

## Figures and Tables

**Table 1:** The physical properties of the tested soil.

Soil Type	Unit Weight/g·cm^−3^	Natural Moisture Content /%	Cohesion /kPa	Internal Friction Angle /°	Liquid Limit /%	Plastic Limit /%	Compression Modulus/MPa
Silt	15.8	14	6	17	30.35	2296	3.6

**Table 2:** Chemical components of the soil sample and the main raw materials of the stabilizer.

	Chemical Components	CaO	SiO_2_	Al_2_O_3_	Fe_2_O_3_	Na_2_O	K_2_O	MgO	SO_3_	P_2_O_5_	Cl
Materials											
Soil		5.79	68.27	11.92	4.30	2.43	2.54	2.23	0.57	0.51	0.02
BFS		40.23	27.25	13.91	0.34	3.97	0.45	8.18	3.69	0.37	0.07
SS		9.66	26.25	6.60	32.13	6.97	2.15	2.20	4.32	0.56	0.14
PG		37.67	7.45	1.17	0.60	0.12	0.40	0.19	50.74	1.19	0.04
OPC		53.8	22.58	7.72	5.26	0.63	1.00	3.34	4.26	0.25	0.13

**Table 3:** The physical properties of the stabilizer.

Materials	Fineness/%	Density /g·cm^−3^	Stability/mm	Setting Time		Fluidity/mm	
				Initial Setting/min	Final Setting/h	Initial	60 min
New soil curing agent	4.8	2.84	2	1.05	8.17	17	39.5

**Table 4:** Variables and experimental plan.

Curing Agent Dosage, *O*_c_/%	Organic Matter Content, *O*_o_/%	Water–Binder Ratio	Curing Age, *T*/d
15, 20, 25, 30	0, 2, 4, 6	0.8	7, 14, 21, 28

**Table 5:** The UCS growth rate of stabilized soil with different dosages of the stabilizer.

Strength Growth Rate	15%~20%				20%~25%				25%~30%			
	0%	2%	4%	6%	0%	2%	4%	6%	0%	2%	4%	6%
7d	44.50%	58.13%	97.24%	384.85%	18.05%	22.98%	39.16%	83.13%	17.84%	24.20%	40.70%	56.66%
14d	38.14%	47.21%	69.67%	93.07%	17.45%	20.70%	29.67%	42.82%	17.08%	23.17%	30.00%	42.01%
21d	34.34%	40.88%	57.38%	71.89%	17.08%	20.00%	22.92%	28.57%	15.79%	18.63%	21.43%	23.96%
28d	32.22%	38.68%	47.14%	54.19%	17.67%	19.31%	21.98%	24.74%	16.70%	18.18%	19.67%	22.25%

**Table 6:** The changes in the strength loss rate as the organic matter content increases.

Strength Loss Rate	2%				4%				6%			
	15%	20%	25%	30%	15%	20%	25%	30%	15%	20%	25%	30%
7d	26.09%	19.12%	15.74%	11.20%	62.92%	49.38%	40.33%	28.75%	91.56%	71.68%	56.07%	41.60%
14d	18.41%	13.05%	10.64%	5.99%	43.07%	30.08%	22.81%	14.29%	61.67%	46.43%	34.85%	20.98%
21d	14.08%	9.89%	7.65%	5.39%	32.44%	20.85%	16.90%	12.86%	46.52%	31.57%	24.85%	19.55%
28d	12.19%	7.90%	6.62%	5.44%	26.42%	18.11%	15.11%	12.95%	37.59%	27.22%	22.85%	19.18%

**Table 7:** The strength growth rate of stabilized soil with curing age.

Strength Growth Rate	7 d~14 d				14 d~21 d				21 d~28 d			
	0%	2%	4%	6%	0%	2%	4%	6%	0%	2%	4%	6%
15%	34.78%	48.79%	106.90%	512.12%	19.92%	26.28%	42.33%	67.33%	9.02%	11.42%	18.74%	27.22%
20%	28.85%	38.51%	77.97%	143.75%	16.62%	20.85%	32.02%	48.97%	7.30%	9.67%	11.01%	14.11%
25%	28.19%	35.94%	65.83%	90.10%	16.26%	20.16%	25.15%	34.11%	7.85%	9.04%	10.17%	10.71%
30%	27.35%	34.81%	53.21%	72.33%	14.99%	15.73%	16.90%	17.07%	8.69%	8.63%	8.57%	9.18%

**Table 8:** The comparison of the previous study and this study.

Reference	Precursors	Stabilizer Content/%	Organic Matter Content/%	Soil Type	UCS/MPa	
					7 Days	28 Days
[37]	The GS binder	16	/	soft clay	/	2.50~3.00
[38]	Early-age strength fly ash-based curing agent	20	/	mucky silty clay	1.20~1.60	1.60~2.0
[39]	Cement, PG, BFS	25	/	soft soil	/	8.85
This study	New stabilizer primarily consists of BFS, SS, PG	20	0	silt	5.65	9.11
[7]	Portland blast furnace cement with a slag content of 65%	20	3.7	silt	0.40~0.50	0.70~0.80
This study	New stabilizer primarily consists of BFS, SS, PG	20	4	silt	2.86	7.46
[40]	A high-efficiency clay stabilizer (cement-based composites, CSCN)	15	5	clay	0.34	0.49
This study	New stabilizer primarily consists of BFS, SS, PG	15	6	silt	0.33	4.30

## Data Availability

All relevant data are available within the article.

## References

[1] Yuan B., Chen W., Zhao J., Yang F., Luo Q., Chen T. (2022). The effect of organic and inorganic modifiers on the physical properties of granite residual soil. Adv. Mater. Sci. Eng..

[2] Yu X., Lu H., Peng J., Ren J., Wang Y., Chen J. (2023). Modified lignin-based cement solidifying material for improving engineering residual soil. Materials.

[3] Zhang X., Ye P., Wu Y., Fujii M., Takahashi A., Wang Y. (2022). Insights into conditioning landfill sludge with freeze-thaw method: Effects on the physical-mechanical properties and micro characteristics. J. Clean. Prod..

[4] Yuan B., Chen W., Li Z., Zhao J., Luo Q., Chen W., Chen T. (2023). Sustainability of the polymer SH reinforced recycled granite residual soil: Properties, physicochemical mechanism, and applications. J. Soils Sediments.

[5] Zhang X., Du D., Wu Y., Ye P., Xu Y. (2024). Theoretical and analytical solution on vacuum preloading consolidation of landfill sludge treated by freeze–thaw and chemical preconditioning. Acta Geotech..

[6] Pan C., Xie X., Gen J., Wang W. (2020). Effect of stabilization/solidification on mechanical and phase characteristics of organic river silt by a stabilizer. Constr. Build. Mater..

[7] Cao Y., Zhang J., Xu G., Li M., Bian X. (2022). Strength properties and prediction model of cement-solidified clay considering organic matter and curing temperature. Front. Mater..

[8] Oliveira P.J.V., Reis M.J. (2023). Effect of the organic matter content on the mechanical properties of soils stabilized with Xanthan gum. Appl. Sci..

[9] Mohammed S.H., Saeed K.A., Al Shaikhli H.I. (2024). Evaluation of the strength and microstructural characteristics of stabilized organic clay soil. Case Stud. Chem. Environ. Eng..

[10] Chen X., Yu F., Hong Z.M., Pan L.F., Liu X.W., Li Y. (2022). Comparative investigation on the curing behavior of GS-stabilized and cemented soils at macromechanical and microstructural scales. J. Test. Eval..

[11] Lang L., Liu N., Chen B. (2022). Investigation on the strength, durability and swelling of cement-solidified dredged sludge admixed fly ash and nano-SiO2. Eur. J. Environ. Civ. Eng..

[12] Sahoo S., Singh S.P. (2022). Strength and durability properties of expansive soil treated with geopolymer and conventional stabilizers. Constr. Build. Mater..

[13] Mohammed A.A., Nahazanan H., Nasir N.A.M., Huseien G.F., Saad A.H. (2023). Calcium-based binders in concrete or soil stabilization: Challenges, problems, and calcined clay as partial replacement to produce low-carbon cement. Materials.

[14] Zhang X., Ye P., Wu Y. (2022). Enhanced technology for sewage sludge advanced dewatering from an engineering practice perspective: A review. J. Environ. Manag..

[15] Peng B., Yang Z., Yang Z., Peng J. (2020). Effects of pH and fineness of phosphogypsum on mechanical performance of cement–phosphogypsum-stabilized soil and classification for road-used phosphogypsum. Coatings.

[16] Samantasinghar S., Singh S.P. (2021). Strength and durability of granular soil stabilized with FA-GGBS geopolymer. J. Mater. Civ. Eng..

[17] Qiu K., Zeng G., Shu B., Luo D. (2023). Study on the performance and solidification mechanism of multi-source solid-waste-based soft soil solidification materials. Materials.

[18] Chen X., Yu F., Yu J., Li S. (2023). Experimental optimization of industrial waste-based soil hardening agent: Combining D-optimal design with genetic algorithm. J. Build. Eng..

[19] Wattez T., Patapy C., Frouin L., Waligora J., Cyr M. (2021). Interactions between alkali-activated ground granulated blastfurnace slag and organic matter in soil stabilization/solidification. Transp. Geotech..

[20] Bian X., Zeng L., Ji F., Xie M., Hong Z. (2022). Plasticity role in strength behavior of cement-phosphogypsum stabilized soils. J. Rock Mech. Geotech. Eng..

[21] Luo Z., Luo B., Zhao Y., Li X., Su Y., Huang H., Wang Q. (2022). Experimental investigation of unconfined compression strength and microstructure characteristics of slag and fly ash-based geopolymer stabilized riverside soft soil. Polymers.

[22] Zheng P., Li W., Ma Q., Xi L. (2023). Mechanical properties of phosphogypsum-soil stabilized by lime activated ground granulated blast-furnace slag. Constr. Build. Mater..

[23] Kumar H., Munisingh M.S. (2024). Development and characterization of fly ash–BFS–cement composite for engineering applications. Int. J. Pavement Res. Technol..

[24] Andavan S., Pagadala V.K. (2020). A study on soil stabilization by addition of fly ash and lime. Mater. Today Proc..

[25] Lang L., Chen B., Chen B. (2021). Strength evolutions of varying water content-dredged sludge stabilized with alkali-activated ground granulated blast-furnace slag. Constr. Build. Mater..

[26] Pu S., Zhu Z., Huo W. (2021). Evaluation of engineering properties and environmental effect of recycled gypsum stabilized soil in geotechnical engineering: A comprehensive review. Resour. Conserv. Recycl..

[27] Yu J., Yu F., Chen X., Li S. (2024). Optimization of mixture ration of industrial solid wastes and cement synergistic solidification engineering waste soil. Sci. Technol. Eng..

[28] He J., Shi X.K., Li Z.X., Zhang L., Feng X.Y., Zhou L.R. (2020). Strength properties of dredged soil at high water content treated with soda residue, carbide slag, and ground granulated blast furnace slag. Constr. Build. Mater..

[29] Mohanty S., Roy N., Singh S.P., Sihag P. (2021). Strength and durability of flyash, GGBS and cement clinker stabilized dispersive soil. Cold Reg. Sci. Tech..

[30] Hu W., Li K., Yin W., Zhang H., Xue Y., Han Y., Liu P. (2024). Effects of wetting–drying cycles on the macro and micro properties of the cement-stabilized soil with curing agent. Buildings.

[31] MolaAbasi H., Semsani S.N., Saberian M., Khajeh A., Li J., Harandi M. (2020). Evaluation of the long-term performance of stabilized sandy soil using binary mixtures: A micro-and macro-level approach. J. Clean Prod..

[32] Wang S., Li X., Ren K., Liu C. (2020). Experimental research on steel slag stabilized soil and its application in subgrade engineering. Geotech. Geol. Eng..

[33] Zhang T., Liu S., Zhan H., Ma C., Cai G. (2020). Durability of silty soil stabilized with recycled lignin for sustainable engineering materials. J. Clean Prod..

[34] Niu W., Guo B., Li K., Ren Z., Zheng Y., Liu J., Men X. (2024). Cementitous material based stabilization of soft soils by stabilizer: Feasibility and durabiliy assessment. Constr. Build. Mater..

[35] (2019). Standard for Geotechnical Testing Method.

[36] (2011). Specification for Mix Proportion Design of Cement Soil.

[37] Ye G., Shu H., Zhang Z., Kang S., Zhang S., Wang Q. (2021). Solidification and field assessment of soft soil stabilized by a waste-based binder using deep mixing method. Bull. Eng. Geol. Environ..

[38] Yang W., Zhou F., Zhu R., Song Z., Hua S., Ma Y. (2022). Strength performance of mucky silty clay modified using early-age fly ash-based curing agent. Case Stud. Constr. Mater..

[39] Dong W., Zhan Q., Zhao X., Wang A., Zhang Y. (2023). Study on the solidification property and mechanism of soft soil based on the industrial waste residue. Rev. Adv. Mater. Sci..

[40] Ma C., Chen B., Chen L. (2016). Effect of organic matter on strength development of self-compacting earth-based construction stabilized with cement-based composites. Constr. Build. Mater..

[41] Häkkinen T. (1993). The influence of slag content on the microstructure, permeability and mechanical properties of concrete Part 1 Microstructural studies and basic mechanical properties. Cem. Concr. Res..

[42] Nidzam R.M., Kinuthia J.M. (2010). Sustainable soil stabilisation with blastfurnace slag–a review. Proceed. Inst. Civ. Eng.-Construct. Mater..

[43] Wu Y., Shi K., Yu J., Han T., Li D. (2020). Research on strength degradation of soil solidified by steel slag powder and cement in seawater erosion. J. Mater. Civ. Eng..

[44] Shen W., Zhou M., Ma W., Hu J., Cai Z. (2009). Investigation on the application of steel slag–fly ash–phosphogypsum solidified material as road base material. J. Hazard. Mater..

[45] Park H., Jeong Y., Jun Y., Jeong J.H., Oh J.E. (2016). Strength enhancement and pore-size refinement in clinker-free CaO-activated GGBFS systems through substitution with gypsum. Cem. Concr. Compos..

[46] Du C., Zhang J., Yang G., Yang Q. (2021). The influence of organic matter on the strength development of cement-stabilized marine soft clay. Mar. Geores. Geotechnol..

[47] Cao J., Liu F., Song Z., Ding W., Guo Y., Li J., Liu G. (2023). Effect of ultra-fine cement on the strength and microstructure of humic acid containing cemented soil. Sustainability.

